# Comparison of the therapeutic effect of Meibomian Thermal Pulsation LipiFlow® on obstructive and hyposecretory meibomian gland dysfunction patients

**DOI:** 10.1007/s10792-020-01533-y

**Published:** 2020-08-01

**Authors:** Bowen Li, Hongxue Fu, Tingting Liu, Mei Xu

**Affiliations:** The First Affiliated Hospital of Chongqing Medical University, Chongqing Key Laboratory of Ophthalmology and Chongqing Eye Institute, Chongqing, China

**Keywords:** Obstructive, Hyposecretory, Meibomian gland dysfunction, Meibomian Thermal Pulsation LipiFlow®

## Abstract

**Purpose:**

To explore the effect of Meibomian Thermal Pulsation LipiFlow® on obstructive and hyposecretory meibomian gland dysfunction.

**Methods:**

Twenty-five subjects diagnosed with obstructive meibomian gland dysfunction (OMGD) and another 25 hyposecretory meibomian gland dysfunction (HMGD) patients were collected receiving the unilateral treatment with LipiFlow®. We evaluated the parameters variables including Standard Patient Evaluation of Eye Dryness (SPEED), Ocular Surface Disease Index (OSDI), Schirmer I test (SIT), noninvasive keratographic breakup time (NIKBUT), tear meniscus height (TMH), and lipid layer thickness (LLT), partial blink rate (PBR), meibomian gland loss, meibomian gland morphology with LipiView®. Meibomian gland expressibility and secretion quality were evaluated for OMGD subjects. All the results were recorded pre-therapy and 4 weeks, 8 weeks, 12 weeks post-therapy.

**Results:**

SPEED, OSDI, and PB decreased, meanwhile, NIKBUT, TMH, SIT, and LLT increased compared with baseline in both groups after treatment (*P* < 0.001), whereas the magnitude of the improvement in the OMGD group was greater than that in the HMGD group (*P* < 0.001). There was no significant posttreatment structural meibomian gland change in both groups. The meibomian gland expressibility and secretion quality score increased after treatment in the OMGD group (*P* < 0.001).

**Conclusions:**

The Meibomian Thermal Pulsation LipiFlow® is effective for both obstructive and hyposecretory meibomian gland dysfunction and the therapeutic effect on obstructive meibomian gland dysfunction is greater than that on hyposecretory meibomian gland dysfunction.

## Introduction

Meibomian gland dysfunction (MGD) is a chronic, diffuse abnormality of the meibomian glands, commonly characterized by terminal duct obstruction and/or qualitative/quantitative changes in the glandular secretion. It may result in alteration of the tear film, symptoms of eye irritation, clinically apparent inflammation and ocular surface disease [1]. It is classified into two major categories based on meibomian gland secretion: low-delivery and high delivery states [1]. Low-delivery states are further classified as hyposecretory meibomian gland dysfunction (HMGD) and obstructive meibomian gland dysfunction (OMGD) [1]. HMGD describes the condition of decreased meibum delivery due to abnormalities in meibomian glands without remarkable obstruction [1, 2]. And OMGD, which is probably the most common form of MGD, is caused by glandular obstruction due to terminal duct obstruction or altered secretion [1, 2]. So, either HMGD or OMGD is characterized by decreased meibum secretion. Meanwhile, the meibum, which is made up of phospholipids, cholesterol, wax esters, cholesterol esters is delivered to the ocular surface to ensure tear film stability [3, 4]. Therefore, lipid abnormalities can lead to the disorder of the tear film composition and function, resulting in evaporative dry eye. There is a strong consensus that MGD is likely the leading cause of dry eye and it can be detected in about 85% of dry eye patients [5]. Thus, the improvement in MGD is a critical success factor for the treatment of dry eye.

Various forms of therapy have been indicated to improve MGD patients’ symptoms and tear film stability, including the use of local artificial tear drops, hormones, antibiotic eye drops and physical methods such as cleansing, warm compresses and massage [6]. Nevertheless, the cornerstone therapy for MGD is the use of warm compresses. In recent years, the Meibomian Thermal Pulsation LipiFlow®, which is a new therapeutic device for MGD that combines the two processes of warm compresses and meibomian gland extrusion, has been put into clinical use [7]. It has been reported that the treatment process is painless with thorough and persistent effects [8]. Several studies have reported that after a single treatment with the meibomian thermal pulsation system, the MGD patients’ ocular discomfort was relieved accompanied by decreased SPEED and OSDI scores and increased tear breakup time [9–11]. However, there was no observation about the therapeutic effect on different types of MGD such as OMGD and HMGD by using the Meibomian Thermal Pulsation LipiFlow®. Thus, we conducted an interventional study using the Meibomian Thermal Pulsation LipiFlow® to treat patients with OMGD and HMGD, respectively, evaluating its therapeutic effects at 4 weeks, 8 weeks and 12 weeks post-therapy.

## Methods

This study was approved by the institutional review board of the First Affiliated Hospital of Chongqing Medical University and conformed to the Helsinki Declaration. In this prospective interventional study, all the participants were recruited from the Department of Ophthalmology, the First Affiliated Hospital of Chongqing Medical University from September 2017 to August 2018. Patients who met the following criteria were enrolled in this study: (1) ≥ 18 years old, (2) symptoms of dry eye (dryness, foreign body sensation, burning, mildly to moderately decreased vision, excess tearing, et al.) more than three months, (3) low tear breakup time (≤ 5 s), (4) low Schirmer I test results (≤ 5 mm), (5) low lipid layer thickness (≤ 60 nm). Patients with pregnancy, lactation, acute inflammation or infection of the eye, the ocular surface disease caused by previous chemical burns or other causes, dry eye syndrome caused by systemic or immune diseases (such as Stevens–Johnson syndrome, rheumatoid arthritis, systemic lupus erythematosus, Sjogren's syndrome, Wegner's granulomatosis, sarcoidosis, leukemia, vitamin A deficiency et al.), history of medications known to cause dry eye syndrome in one month (e.g., antihistamines, etc.), history of eye surgery within 3 months (especially lacrimal punctum occlusion or lacrimal duct recanalization), participation in other ophthalmic clinical studies 30 days before the baseline examination were excluded.

### Categorization criteria

According to the classification criteria mentioned above, participants were categorized into two groups: OMGD and HMGD. 25 participants with OMGD were recruited (8 males and 17 females; mean age: 37.2 ± 11.74 years; range 21–64 years). At the same time, twenty-five eyes of 25 age- and gender-matched patients with HMGD (5 males and 20 females; mean age: 32.6 ± 10.26 years; range 19–57 years) were also included in the study. All subjects’ right eyes were selected.

### Questionnaires for dry eye symptoms

The Standard Patient Evaluation of Eye Dryness (SPEED) and the Ocular Surface Disease Index (OSDI) questionnaire were both applied for the assessment of ocular symptoms. The SPEED questionnaire had been developed to assess the frequency and severity of dry eye symptoms, especially for the assessment of longer-term symptom changes over 3 months with a score ranging from 0 to 28 [12]. The Ocular Surface Disease Index (OSDI) was designed to evaluate the frequency of symptoms over the preceding week, and the scores ranged from 0 to 100 [13]. It should be noted that the higher the scores of either of the two questionnaires were, the more severe the symptoms were.

### NIKBUT, TMH

Noninvasive keratographic breakup time (NIKBUT) and tear meniscus height (TMH) were evaluated automatically through a Keratograph 5M (OCULUS, Wetzlar, Germany) [14, 15]. Each patient was asked to blink three times, then, to keep their eyes open as long as possible. The time between the last blink and the first sign of distortion of the ring pattern was recorded. The average of the three measurements was recorded as the final NIKBUT. TMH was also measured three times for 3 s at the inferior of the cornea with modified tear film scanning function.

### Schirmer I test (SIT)

The tear strip (30 mm; Jingming Tianjin, China) was folded in the front, placed in the mid-lateral portion of the lower fornix. Then patients were asked to close their eyes. The length of the wetting strip was recorded after 5 min.

### LLT, partial blink rate, and meibography

LipiView® II (TearScience, Inc., Morrisville, NC) was applied to measure lipid layer thickness (LLT) of the tear film, calculate partial blink rate (PBR) and image the meibomian gland [16, 17]. Patients were instructed to look straight ahead blinking normally in 20 s of the monocular measurement process. The results of LLT and PBR were automatically obtained after the measurement. Meibography images of the upper and lower eyelids were performed to assess the structure of the meibomian gland after satisfying focus. The meibomian gland loss was analyzed by using ImageJ as described by Pult and Nichols [18].

### Meibomian gland expressibility and secretion quality

The Meibomian Gland Evaluator (MGE, TearScience, Inc.) was used to simulate the constant, gentle pressure (1.2 g/mm^2^) on the participant’s lower eyelid, similar to the pressure of a normal blink, and then, the meibomian gland orifices and secretions were observed through the slit-lamp microscope. The MGE was placed in the lower eyelid 2 mm from the root of the eyelashes, 45° upward, and maintained 10–15 s. Five glands were evaluated, respectively, in the temporal, central, and nasal parts of the lower eyelid, 15 glands in total. The assessment of secretion quality of each of the 15 glands in the lower eyelid was scored 0–3, corresponding to no secretion (grade 0), inspissated secretion (grade 1), cloudy secretion (grade 2), clear secretion (grade 3), with total scores 45 points [11]. The examination was exclusively completed in the OMGD group.

### Treatment by Meibomian Thermal Pulsation LipiFlow®

Before treatment, the patients were asked to take a comfortable position. Anesthetic drops were dripped down in the conjunctival sac two times and the eyelid heater was placed on the surface of the palpebral conjunctiva of the subject, warming up for 12 min, keeping the temperature at 42.5 °C. The eyecup was located on the surface of the eyelid, and then, the meibomian gland was extruded by a constant pressure (pulsing pressure < 41 kPa) along the direction of the meibomian gland orifices [19]. As a result, the dissolved meibum was discharged.

### Statistical analysis

SPSS software (SPSS, version 20.0, Inc., USA) was used for statistical analysis. All the results were described as mean ± standard deviation. Paired samples T test was applied to compare the differences of the parameters before and after therapy of the two groups. Independent samples *T* test was used to compare the differences of the parameters between the OMGD group and the HMGD group. The correlation between parameters was assessed by Pearson's correlation coefficient. A *P* value < 0.05 was considered statistically significant.

## Results

All 50 participants (50 eyes) in this study completed the treatment successfully and followed up for 12 weeks.

### Ocular symptoms

The SPEED and OSDI scores in both OMGD and HMGD group significantly decreased at 4 weeks, 8 weeks, 12 weeks compared with baseline (Tables 1, 2). The SPEED scores between the OMGD and HMGD group were statistically different at 4 weeks (7.72 ± 2.26, 8.92 ± 2.14, *P* < 0.001), 8 weeks (4.12 ± 2.15, 6.64 ± 1.68, *P* < 0.001) and 12 weeks (1.52 ± 1.26, 5.20 ± 1.96, *P* < 0.001). Moreover, there was difference in OSDI scores between the OMGD and HMGD group at 4 weeks (7.61 ± 2.33, 17.31 ± 4.71, *P* < 0.001), 8 weeks (3.69 ± 1.43, 13.86 ± 4.21, *P* < 0.001), and 12 weeks (1.74 ± 0.77, 12.92 ± 4.12, *P* < 0.001).

**Table 1:** The results before and 4, 8, 12 weeks after treatment with LipiFlow® in the OMGD group

Project	*N*	Baseline	4 weeks			8 weeks			12 weeks		
		$\overline{x} \pm s$	$\overline{x} \pm s$	*t*	*P*_4_	$\overline{x} \pm s$	*t*	*P*_8_	$\overline{x} \pm s$	*t*	*P*_12_
SPEED	25	11.96 ± 2.46	7.72 ± 2.26	18.787	< 0.001*	4.12 ± 2.15	34.293	< 0.001*	1.52 ± 1.26	27.55	< 0.001*
OSDI	25	22.91 ± 4.42	7.61 ± 2.33	25.355	< 0.001*	3.69 ± 1.43	28.362	< 0.001*	1.74 ± 0.77	26.623	< 0.001*
SIT/mm	25	1.76 ± 1.09	6.16 ± 1.91	− 11.237	< 0.001*	4.68 ± 1.60	− 9.405	< 0.001*	3.96 ± 1.62	− 6.264	< 0.001*
NIKBUT/s	25	3.22 ± 1.47	10.97 ± 2.23	− 21.081	< 0.001*	8.13 ± 2.05	− 11.98	< 0.001*	4.37 ± 1.37	− 3.405	0.002*
TMH/mm	25	0.19 ± 0.11	0.32 ± 0.12	− 11.371	< 0.001*	0.28 ± 0.11	− 9.388	< 0.001*	0.25 ± 0.11	− 6.998	< 0.001*
LLT/nm	25	44.72 ± 10.01	75.52 ± 12.53	− 18.212	< 0.001*	68.64 ± 13.10	− 14.99	< 0.001*	61.52 ± 11.82	− 11.422	< 0.001*
PBR	25	0.58 ± 0.16	0.45 ± 0.15	14.147	< 0.001*	0.35 ± 0.12	16.545	< 0.001*	0.36 ± 0.12	13.573	< 0.001*

$\overline{x} \pm s$: mean ± standard deviation; *P*_4_: *P* value at 4 weeks from baseline; *P*_8_: *P* value at 8 weeks from baseline; *P*_12_: *P* value at 12 weeks from baseline

*SPEED* Standard Patient Evaluation of Eye Dryness, *OSDI* Ocular Surface Disease Index, *SIT* Schirmer I test, *NIKBUT* noninvasive keratographic breakup time, *TMH* tear meniscus height, *LLT* lipid layer thickness, *PBR* partial blink rate

*Statistically significant

**Table 2:** The results before and 4, 8, 12 weeks after treatment with LipiFlow® in the HMGD group

Project	*N*	Baseline	4 weeks			8 weeks			12 weeks		
		$\overline{x} \pm s$	$\overline{x} \pm s$	*t*	*P*_4_	$\overline{x} \pm s$	*t*	*P*_8_	$\overline{x} \pm s$	*t*	*P*_12_
SPEED	25	12.12 ± 2.45	8.92 ± 2.14	13.443	< 0.001*	6.64 ± 1.68	16.784	< 0.001*	5.20 ± 1.96	18.086	< 0.001*
OSDI	25	24.12 ± 4.84	17.31 ± 4.71	2.394	< 0.001*	13.86 ± 4.21	14.276	< 0.001*	12.92 ± 4.12	15.95	< 0.001*
SIT/mm	25	1.48 ± 1.00	3.88 ± 1.17	− 7.719	< 0.001*	2.88 ± 1.27	− 4.427	< 0.001*	2.00 ± 1.35	− 1.641	0.114
NIKBUT/s	25	3.17 ± 1.40	5.49 ± 1.57	− 7.653	< 0.001*	3.77 ± 1.27	− 2.902	0.008*	2.95 ± 1.14	1.208	0.239
TMH/mm	25	0.19 ± 0.09	0.24 ± 0.09	− 19.092	< 0.001*	0.22 ± 0.09	− 10.533	< 0.001*	0.20 ± 0.09	− 4.301	< 0.001*
LLT/nm	25	44.40 ± 9.45	46.44 ± 9.79	− 8.981	< 0.001*	45.32 ± 9.75	− 2.326	0.029*	44.72 ± 9.16	− 0.969	0.342
PBR	25	0.61 ± 0.19	0.51 ± 0.17	13.843	< 0.001*	0.42 ± 0.15	16.794	< 0.001*	0.42 ± 0.14	13.913	< 0.001*

$\overline{x} \pm s$: mean ± standard deviation; *P*_4_: *P* value at 4 weeks from baseline; *P*_8_: *P* value at 8 weeks from baseline; *P*_12_: *P* value at 12 weeks from baseline

*SPEED* Standard Patient Evaluation of Eye Dryness, *OSDI* Ocular Surface Disease Index, *SIT* Schirmer I test, *NIKBUT* noninvasive keratographic breakup time, *TMH* tear meniscus height, *LLT* lipid layer thickness, *PBR* partial blink rate

*Statistically significant

### NIKBUT, TMH

The mean NIKBUT increased at 4 weeks and gradually decreased at 8 weeks, 12 weeks in both groups (Tables 1, 2). The mean NIKBUT at different time points significantly improved compared with baseline in the OMGD group (Table 1). However, in the HMGD group, it significantly improved at 4 weeks, 8 weeks compared with baseline, but not statistically different from baseline at 12 weeks (Table 2). The NIKBUT between the OMGD and HMGD group were statistically different at 4 weeks (10.97 ± 2.23, 5.49 ± 1.57, *P* < 0.001), 8 weeks (8.13 ± 2.05, 3.77 ± 1.27, *P* < 0.001) and 12 weeks (4.37 ± 1.37, 2.95 ± 1.14, *P* < 0.001).

The mean TMH also increased at 4 weeks and gradually decreased at 8 weeks, 12 weeks with all results significantly increased from baseline in both groups (all *P* < 0.001) (Tables 1, 2). There was a significant difference in TMH between the two groups at 4 weeks (0.32 ± 0.12, 0.24 ± 0.09, *P* < 0.001).

### Schirmer I test (SIT)

Schirmer I test improved significantly in both groups at 4 weeks from baseline and gradually decreased at 8 weeks and 12 weeks (Tables 1, 2). In the OMGD group, SIT also improved significantly at 8 weeks, 12 weeks compared with baseline (Table 1). In the HMGD group, SIT improved at 8 weeks from baseline, but it was not significantly different at 12 weeks (Table 2). When undergoing treatment, there was a significant difference between the OMGD and HMGD group at 4 weeks (6.16 ± 1.91, 3.88 ± 1.17, *P* < 0.001), 8 weeks (4.68 ± 1.60, 2.88 ± 1.27, *P* < 0.001) and 12 weeks (3.96 ± 1.62, 2.00 ± 1.35, *P* < 0.001).

### LLT

LLT reached a peak at 4 weeks and gradually decreased at 8 weeks, 12 weeks in both groups (Tables 1, 2). In the OMGD group, all results significantly increased from baseline (Table 1). In the HMGD group, LLT also significantly increased at 4 weeks, 8 weeks but it was not significantly different at 12 weeks compared with baseline (Table 2). There was a significant difference between the two groups at 4 weeks (75.52 ± 12.53, 46.44 ± 9.79, *P* < 0.001), 8 weeks (68.64 ± 13.10, 45.32 ± 9.75, *P* < 0.001) and 12 weeks (61.52 ± 11.82, 44.72 ± 9.16, *P* < 0.001).

### Blinking pattern

In both groups, the partial blink rate gradually decreased, statistically significant from baseline (Tables 1, 2). No significant difference between the two groups at 4 weeks (0.45 ± 0.15, 0.51 ± 0.17, *P* = 0.242), 8 weeks (0.35 ± 0.12, 0.42 ± 0.15, *P* = 0.108) and 12 weeks (0.36 ± 0.12, 0.42 ± 0.14, *P* = 0.104) was noted.

### Meibomian gland loss

**Fig. 1:**
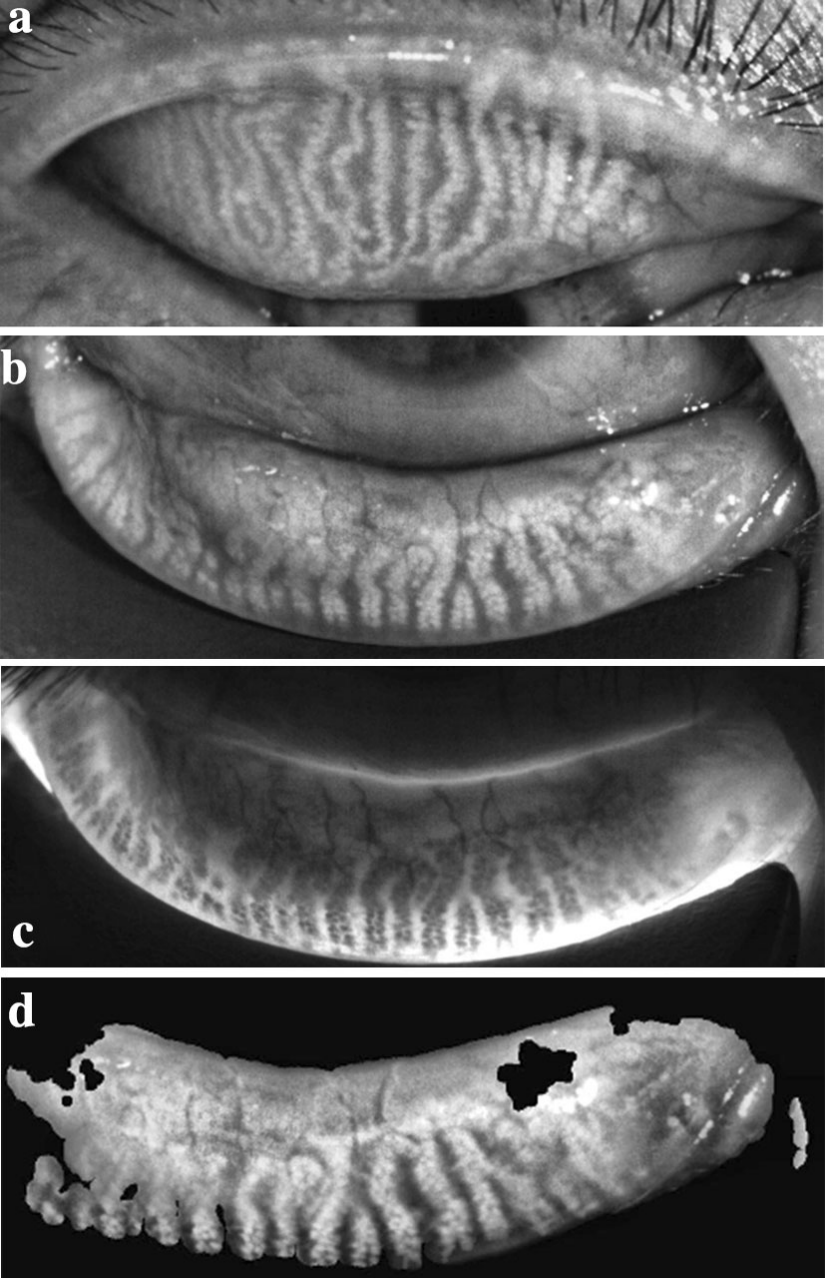
Meibography of an OMGD patient with meibomian gland distortion and loss

**Fig. 2:**
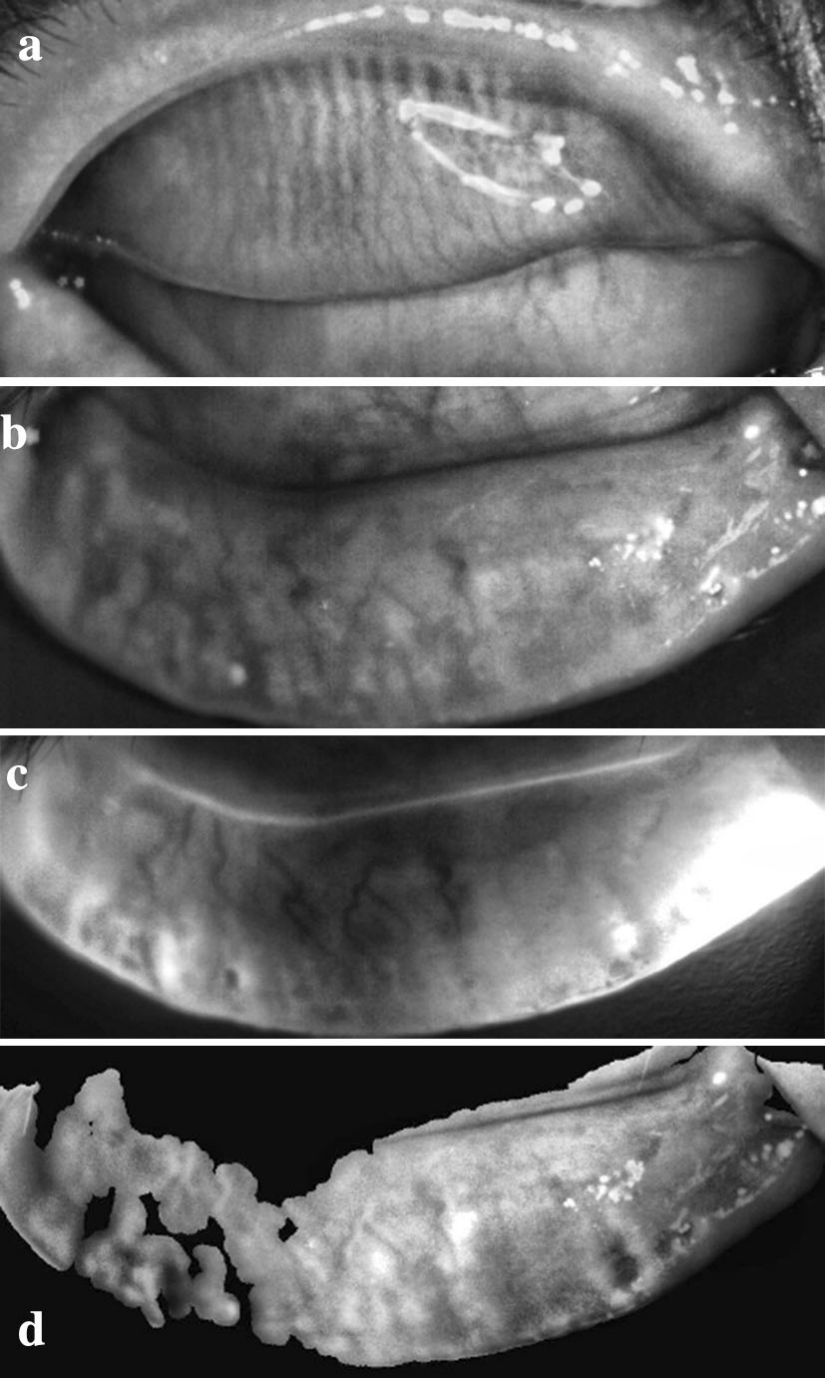
Meibography of an HMGD patient with severe meibomian gland atrophy and loss

The grade of meibomian gland loss was divided into 4 levels: 0% (grade 0), < 33% (grade 1), 33–67% (grade 2), and > 67% (grade 3) [14]. The gland loss rate of the lower tarsus was more severe than that of the upper one in both groups (Tables 3, 4). No tarsus degeneration was found, and the loss rate of the meibomian gland did not further increase during the 12-week follow-up period (Figs. 1, 2).

**Table 3:** Meibomian gland loss of the upper eyelid in OMGD and HMGD subjects

Degree	OMGD	HMGD
0	0% (0/25)	0% (0/25)
1	64% (16/25)	52% (13/25)
2	36% (9/25)	44% (11/25)
3	0% (0/25)	4% (1/25)

**Table 4:** Meibomian gland loss of the lower eyelid in OMGD and HMGD subjects

Degree	OMGD	HMGD
0	0% (0/25)	0% (0/25)
1	48% (12/25)	40% (10/25)
2	44% (11/25)	52% (13/25)
3	4% (1/25)	8% (2/25)

### Meibomian gland expressibility and secretion quality

**Fig. 3:**
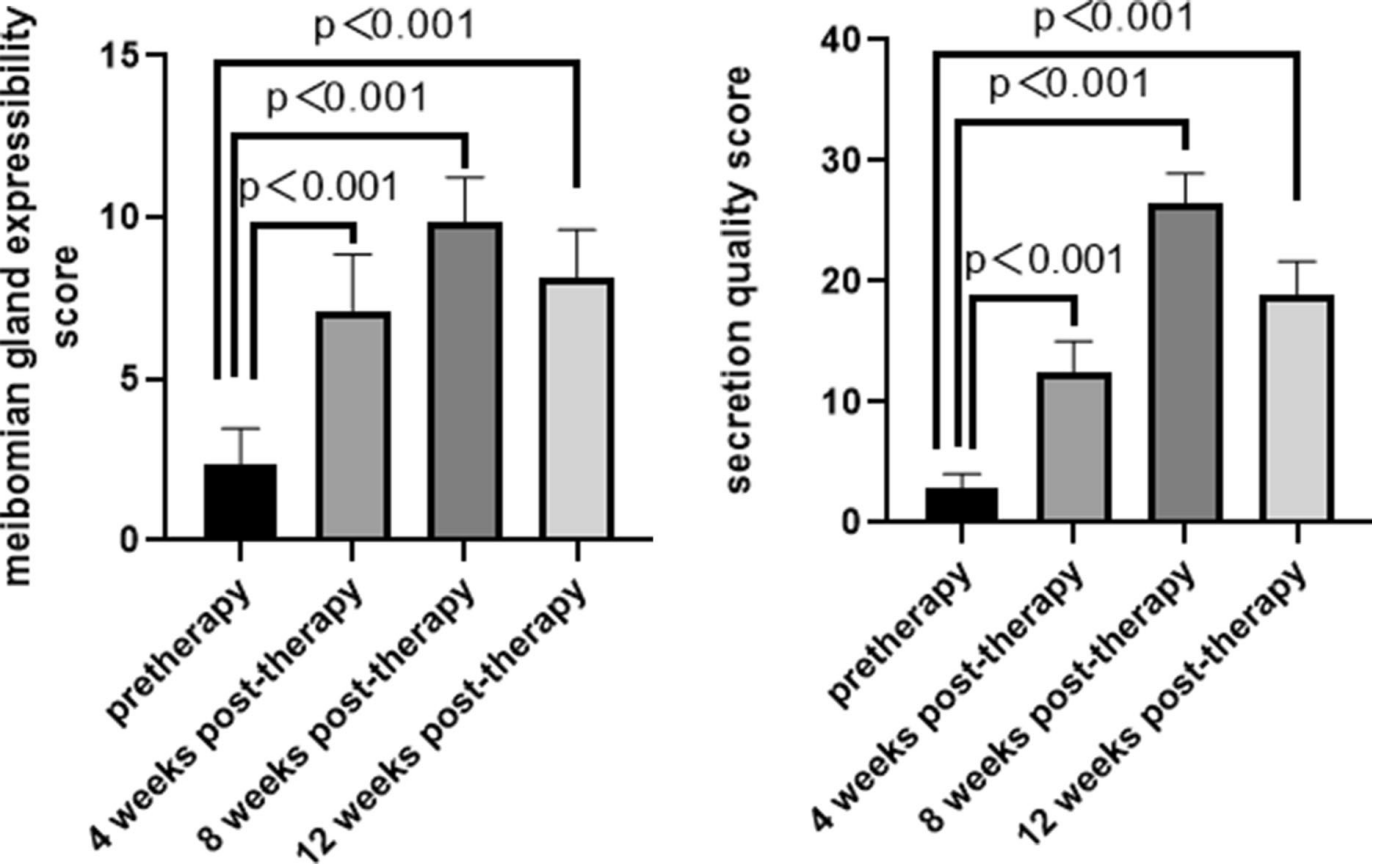
Meibomian gland expressibility and secretion quality scores in OMGD patients before and after a single LipiFlow® treatment

The meibomian gland expressibility and secretion quality in the OMGD group improved post-therapy and reached a peak at 8 weeks. (Fig. 3).

### Correlation analysis

In the OMGD group, we found a positive correlation between LLT and meibomian gland expressibility score at 4 weeks, 8 weeks, 12 weeks, and NIKBUT was positively correlated with LLT at 4 weeks, 8 weeks, 12 weeks. Moreover, in the OMGD group, there was a significantly positive correlation between meibomian gland expressibility score and secretion quality score at 8 weeks, 12 weeks (Table 5). In the HMGD group, NIKBUT positively correlated with LLT at any point (Table 6).

**Table 5:** The correlation between parameters in the OMGD group

Project	Baseline		4 weeks		8 weeks		12 weeks	
	*r*	*P*_0_	*r*	*P*_4_	*r*	*P*_8_	*r*	*P*_12_
LLT-SPEED	− 0.681	< 0.001*	− 0.423	0.035*	− 0.554	0.004*	− 0.153	0.466
LLT-OSDI	− 0.673	< 0.001*	− 0.793	< 0.001*	− 0.609	0.001*	− 0.56	0.004*
LLT-NIKBUT	0.66	< 0.001*	0.702	< 0.001*	0.563	0.003*	0.496	0.012*
LLT-SIT	0.322	0.117	0.387	0.056	0.37	0.069	0.423	0.035*
PBR-LLT	− 0.726	< 0.001*	− 0.812	< 0.001*	− 0.756	< 0.001*	− 0.714	< 0.001*
MGE-LLT	0.118	0.575	0.551	0.004*	0.41	0.042*	0.665	< 0.001*
SQ-LLT	0.576	0.003*	0.452	0.023*	0.603	0.001*	0.431	0.032*
MGE-SQ	− 0.009	0.964	0.152	0.469	0.623	0.001*	0.686	< 0.001*

*SPEED* Standard Patient Evaluation of Eye Dryness, *OSDI* Ocular Surface Disease Index, *SIT* Schirmer I test, *NIKBUT* noninvasive keratographic breakup time, *LLT* lipid layer thickness, *PBR* partial blink rate, *MGE* meibomian gland expressibility, *SQ* secretion quality

*Statistically significant

**Table 6:** The correlation between parameters in the HMGD group

Project	Baseline		4 weeks		8 weeks		12 weeks	
	*r*	*P*_0_	*r*	*P*_4_	*r*	*P*_8_	*r*	*P*_12_
LLT-SPEED	− 0.66	< 0.001*	− 0.603	0.001*	− 0.532	0.006*	− 0.45	0.024*
LLT-OSDI	− 0.45	0.024*	− 0.199	0.341	− 0.054	0.796	− 0.139	0.506
LLT-NIKBUT	0.697	< 0.001*	0.463	0.02*	0.758	< 0.001*	0.648	< 0.001*
LLT-SIT	− 52	0.806	0.538	0.006*	0.491	0.012*	0.635	0.001*
PBR-LLT	− 783	< 0.001*	− 0.769	< 0.001*	− 0.719	< 0.001*	− 0.692	< 0.001*

*SPEED* Standard Patient Evaluation of Eye Dryness, *OSDI* Ocular Surface Disease Index, *SIT* Schirmer I test, *NIKBUT* noninvasive keratographic breakup time, *LLT* lipid layer thickness, *PBR* partial blink rate

*Statistically significant

## Discussion

The prevalence of MGD varies widely, from 3.5% to almost 70% and increases year by year [20]. MGD is likely the leading cause of evaporative dry eye. It is reported that compromise to meibomian gland function negatively impacts all aspects of ocular surface health and effective therapy for MGD will bring significant improvement to dry eye syndrome [3]. There are many treatments for MGD. Physical therapy is one of the most critical approaches, such as eyelid cleansing, warm compresses, meibomian gland massage, intense pulsed laser therapy, treatment with thermal pulsation therapeutic device and fiber meibomian gland probe, etc. [6].

In recent years, Meibomian Thermal Pulsation LipiFlow® (TearScience Inc., Morrisville, NC, USA), as a promising physical therapy for MGD, applies heat (42.5 °C) to both inner eyelid surfaces, insulating the eye from the heat while pulsating pressure is simultaneously applied to the outer eyelids using an inflatable air bladder [7]. This temperature allows to unblock glandular plugging by dissolving the meibum effectively and facilitates the meibum to discharge easily [21]. At the same time, the temperature will not cause thermal injuries and the constant pressure squeezes the meibomian gland promoting the discharge of melted meibum with less discomfort compared with the pain caused by manual extrusion [22]. Moreover, both eyes could be simultaneously treated and it has been demonstrated that a single LipiFlow® treatment would sustain efficaciously for months in patients with MGD [11].

There are existing researches that have been conducted for the therapeutic effect of LipiFlow® on MGD [7–11], but among which there is no published report about the effect on different types of MGD. In this study, we found that a single LipiFlow® treatment was effective on both groups. All the patients experienced symptomatic improvement, and NIKBUT, TMH, LLT significantly increased. These are consistent with the previous reports [8, 9, 23, 24]. However, the difference in the therapeutic effects between the OMGD and HMGD group was detected.

In our study, as for ocular symptoms, although the posttreatment improvement sustained up to 12 weeks in both groups, the improvement degree of the HMGD group was not as much as that of the OMGD group, and the difference was statistically significant. As for the objective ocular signs, in the OMGD group, the significant increase in NIKBUT and SIT sustained up to 12 weeks. However, in the HMGD group, they only lasted for 8 weeks. Compared with the HMGD group, the improvement in NIKBUT, SIT and TMH tended to be greater at any point after treatment in the OMGD group in our study. In terms of LLT, in the OGMD group, it increased significantly and sustained to 12 weeks. While in the HMGD group, compared with the OMGD group, the posttreatment increase in LLT was smaller and not clinically significant at 12 weeks from baseline.

Thus, we speculate that the symptomatic relief and glandular functional improvement in the OMGD group were more effective than in the HMGD group. In the OMGD group, the therapeutic effect might be due to the evacuation of gland contents that improved the quantity and quality of the meibum which plays an important role in tear film stabilization. This effect was probably attributed to the design of the LipiFlow® device which combines the eyelid heating and the mechanical massage of the gland from pulsating pressure on the outer eyelid, offering effective therapy for meibomian gland obstruction [7]. The blocked meibomian gland orifices were recanalized and the stagnated contents in meibomian glands were evacuated. As a result, the amount of meibum on the ocular surface increased, stabilizing the tear film and relieving ocular discomfort. As the number of recanalized orifices increasing, the number of glands that could discharge meibum also increased and the meibum quality improved, from no secretion or inspissated secretion to cloudy secretion even clear secretion. Finally, symptoms and signs were improved effectively in the OMGD group.

In the HMGD group, our results indicated that the meibomian gland function was also improved after a single LipiFlow® treatment, but the effect sustained up to a period at most 8 weeks during our follow-up, shorter than the OMGD group. However, the subjective symptomatic improvement in HMGD patients lasted for 12 weeks in our study. This trend was similar to the previous study which showed a significant posttreatment improvement in symptoms at 1 month with sustained effects at 3 months and the improvement in the clinical sign at 1 month was significant but not sustained to 3 months [19]. In response to these results, we presumed that a small amount of meibum residue in glands, although without gland obstruction, could also be discharged after the treatment of LipiFlow®. However, the effect was relatively small compared with OMGD patients. Moreover, we considered that the mainly active treatment on HMGD might be attributed to the improvement in meibomian gland secretion by heating and massage simulation.

It should be noted that the stagnated meibum in the OMGD group may accumulate in the glandular duct over time blocking the duct once again. Because the NIKBUT, LLT started to decrease at 8 weeks and the meibomian gland expressibility and secretion quality score started to decrease at 12 weeks. A longer follow-up for OMGD is necessary to observe the time point when the parameters return to baseline. A previous study reported that the decreased SPEED and the increased meibomian gland secretion score could sustain up to 3 years [8]. In our study in the HMGD group, the improved meibomian gland secretion lasts for only 8 weeks during our follow-up. Therefore, if glandular obstruction reappears or meibomian gland secretion decreases, the LipiFlow® treatment may be applied again in time to restore the smooth discharge and improve the meibomian gland function.

With respect to our exploratory outcomes, we found the partial blink rate decreased after treatment and there was no statistical difference in both groups. The decreased partial blink rate was probably due to patients’ subjective training in blinking. Complete blink probably helps to form a full tear film on the ocular surface.

The improvement in the meibomian gland function was inconsistent with the meibomian gland structure in our study. Different grades of meibomian gland loss were found in all fifty eyes and no structural meibomian gland regeneration or increased meibomian gland loss was observed during our follow-up. Maybe a longer follow-up duration is necessary for the assessment of structural and functional change of meibomian glands. Previous studies have confirmed that meibomian gland loss is associated with age, gender, etc. [5, 25, 26]. In some cases of primary meibomian gland loss, the loss rate of the superior meibomian gland was lower than that of the inferior gland [23]. The superior meibomian gland discharges the meibum smoothly by gravity, however, the inferior meibum discharge is in the opposite direction of gravity which is more likely to cause the glandular duct obstruction and eventually lead to meibomian gland atrophy and loss [23]. Some prior studies also found that the lateral meibomian gland loss was more severe than the central gland loss and MGD occurred more often in the nasal side [27, 28].

There is a limitation to our study. We have only 25 participants in each group and 12 weeks of follow-up. A large-scale case and longer follow-up are necessary for future studies. Nevertheless, the issue of greater concern is that there is a therapeutic difference between the OMGD and HMGD group.

In conclusion, this study demonstrated that Meibomian Thermal Pulsation LipiFlow®, currently a novel physical therapy for MGD, achieved symptomatic relief and improvement in meibomian gland function in both OMGD and HMGD group after a single treatment. However, the magnitude of the improvement in the OMGD group was greater than that in the HMGD group. An observation in the structural change of the meibomian gland in both groups is required in the subsequent study.
